# *ShORR-1*, a Novel Tomato Gene, Confers Enhanced Host Resistance to *Oidium neolycopersici*

**DOI:** 10.3389/fpls.2019.01400

**Published:** 2019-11-07

**Authors:** Yi Zhang, Kedong Xu, Dongli Pei, Deshui Yu, Ju Zhang, Xiaoli Li, Guo Chen, Hui Yang, Wenjie Zhou, Chengwei Li

**Affiliations:** ^1^Key Laboratory of Plant Genetics and Molecular Breeding, Zhoukou Normal University, Zhoukou, China; ^2^Henan Key Laboratory of Crop Molecular Breeding & Bioreactor, Zhoukou, China; ^3^Department of Life Science, Shangqiu Normal University, Shangqiu, China; ^4^Henan Engineering Research Center of Grain Crop Genome Editing, Henan Institute of Science and Technology, Xinxiang, China

**Keywords:** *Oidium neolycopersici*, *Ol-1*-mediated resistance, susceptible tomato, resistant tomato, H_2_O_2_ accumulation, abnormal haustoria

## Abstract

A previous complementary cDNA-amplified fragment length polymorphism (cDNA-AFLP) analysis examined responses to the powdery mildew pathogen *Oidium neolycopersici* (*On*) of the resistant cultivar *Solanum habrochiates* G1.1560, carrying the *Ol-1* resistance gene, and susceptible cultivar *S. lycopersicum* Moneymaker (MM). Among other findings, a differentially expressed transcript-derived fragment (DE-TDF) (M14E72-213) was upregulated in near isogenic line (NIL)-*Ol-1*, but absent in MM. This DE-TDF showed high homology to a gene of unknown function, which we named *ShORR-1* (*Solanum habrochaites Oidium Resistance Required-1*). However, MM homolog of *ShORR-1* (named *ShORR-1-M*) was still found with 95.26% nucleic acid sequence similarity to *ShORR-1* from G1.1560 (named *ShORR-1-G*); this was because the cut sites of restriction enzymes in the previous complementary cDNA-AFLP analysis was absent in *ShORR-1-M* and differs at 13 amino acids from *ShORR-1-G*. Transient expression in onion epidermal cells showed that ShORR-1 is a membrane-localized protein. Virus-induced gene silencing (VIGS) of *ShORR-1-G* in G1.1560 plants increased susceptibility to *On*. Furthermore, overexpressing of *ShORR-1-G* conferred MM with resistance to *On*, involving extensive hydrogen peroxide accumulation and formation of abnormal haustoria. Knockdown of *ShORR-1-M* in MM did not affect its susceptibility to *On*, while overexpressing of *ShORR-1-M* enhanced MM’s susceptibility to *On*. We also found that changes in transcript levels of six well-known hormone signaling and defense-related genes are involved in *ShORR-1-G*-mediated resistance to *On*. The results indicate that *ShORR-1-M* and *ShORR-1-G* have antagonistic effects in tomato responses to *On*, and that *ShORR-1* is essential for *Ol-1*-mediated resistance in tomato.

## Introduction

Plants have evolved a multilayered immune system that prevents or hinders colonization by most potential pathogens. To date, two types of innate immune response have been recognized in plants. One is pathogen-associated molecular pattern-triggered immunity, which is activated by a number of pathogen-associated molecular patterns such as flagellin, EF-Tu, and chitin, and perceived by pattern recognition receptors (Jones and Dangl, 2006; Dodds and Rathjen, 2010; Peng et al., 2018). The other is effector-triggered immunity, which is modulated by recognition of pathogen-derived avirulence effectors by plant R proteins (Jones and Dangl, 2006; Dodds and Rathjen, 2010).

Powdery mildew caused by *Oidium neolycopersici* (*On*) is one of the most severe diseases of tomato. Six resistance genes (termed *Ol-X*) and three quantitative resistance loci from wild tomato species have been identified, all of which mediate various resistance responses to *On* (Bai et al., 2003; Bai et al., 2004; Bai et al., 2005; Li, 2005). *Ol-1*, one of the resistance genes, derived from *Solanum habrochaites* G1.1560 (Lindhout et al., 1994), mediates partial resistance to *On*, including a slow hypersensitive response (HR) (Li et al., 2007). *Ol-4*, introgressed from wild tomato species *S. peruvianum* LA2172, confers complete resistance to *On* and is associated with a rapid HR (Bai et al., 2004). In previous studies, we found that near-isogenic lines (NILs) carrying *ol-2*, *Ol-1* and *Ol-4* genes in the genetic background of the susceptible cultivar MM had varying degrees of resistance to *On* (Li et al., 2006; Li et al., 2007). We also examined gene expression patterns in these lines by cDNA-amplified fragment length polymorphism (cDNA-AFLP) analysis. Transcript-derived fragments (TDFs) showing differential presence or intensity between resistant tomato NILs and susceptible MM after mock-inoculation and inoculation with *On* were identified (Li et al., 2006; Li et al., 2007). UniGene sequences in the Solanaceae Genomics Network (SGN) database showing high homology to each differentially expressed TDF were then identified. Tobacco rattle virus (TRV)-based virus-induced gene silencing (VIGS) constructs targeting these UniGenes were subsequently generated, and used to determine whether silencing the targeted genes altered the *On* resistance of relevant genotypes. These efforts revealed that acetolactate synthase (ALS) (Gao et al., 2014), a glutathione S-transferase (GST) gene (Pei et al., 2011a), and an NADP-malic enzyme gene (Pei et al., 2011b) are required for *Ol-1*-mediated resistance to *On*.

In the study presented here, we focused on another differentially expressed TDF (M14E72-213) and analyzed its involvement in *On* resistance. M14E72-213 is present in NIL-*Ol-1*, but not MM or NIL-*Ol-4* (Li et al., 2006; Li et al., 2007). We found that silencing it resulted in loss of resistance, to varying degrees, in *S. habrochaites* G1.1560 (which carries the *Ol-1* gene). Microscopic observation showed that the pathogen could complete its life cycle on leaves of the plants with silenced *Ol-1*. In addition, the HR was slow in epidermal cells of the leaves from this line, while rapid HR in attacked epidermal cells of control plants prevented completion of *On*’s life cycle. Thus, the gene is apparently required for *Ol-1* mediated resistance to *On*, and was named *ShORR-1* (*S. habrochaites Oidium Resistance Required gene*). According to open reading frame (ORF) finder, it encodes a putative 268 amino acid protein, which has 93% identity with an uncharacterized gene (XM_004242006), suggesting that it may be a novel gene. The results indicated that differences in *ShORR-1* variants of susceptible and resistant tomato account for at least some of the differences in their resistance. Further analyses indicate that increases in H_2_O_2_ accumulation and formation of abnormal haustoria are involved in *Ol-1*-mediated resistance to powdery mildew in tomato, which requires an appropriate variant of *ShORR-1*, such as *ShORR-1-G*.

## Materials and Methods

### Plant Materials, Pathogen, Inoculation, and Treatments

The *On-*susceptible tomato *S. lycopersicum* Mill (MM) and resistant cultivar *S. habrochaites* G1.1560, which carries the *Ol-1* gene, were grown in a greenhouse providing 16 h and 23°C day/8 h and 20°C night cycles, with constant 80% relative humidity (RH). The tomato powdery mildew used in this research was identified as *On* isolate China (Li et al., 2008) on the basis of its morphological, histological, and molecular characteristics. The fungus was maintained on the susceptible tomato cultivar MM. For inoculation of *On*, whole plants were sprayed with a suspension of spores (5 × 10^3^ conidia/ml) collected from infected tomato plants in a climate chamber in the conditions mentioned above, except that the RH was 85 and 95% during the day and night periods, respectively. Control plants were sprayed with sterile water.

### Vector Construction and Virus-Inducing Gene Silencing Assays

The target DE-TDF was amplified by reverse transcription PCR (RT-PCR) with primers listed in **Table 1**. The recombinant vector TRV-LIC-*ShORR-1* carrying the target sequence was constructed as described elsewhere (Dong et al., 2007). The vectors TRV1 and TRV2-LIC-*ShORR-1* were introduced into *Agrobacterium tumefaciens* strain GV3101 by heat shock and cocultured overnight. Overnight cultures (5 ml) were grown at 28°C in appropriate antibiotic selection medium in 15 ml glass tubes for 1 day, then briefly centrifuged, and the collected cells were resuspended in infiltration medium (10 mM MES, 10 mM MgCl_2_, 200 µM acetosyringone) to an OD_600_ of 1. After incubation at room temperature for 3 h, the cultures were used for agro-infiltration, as previously reported (Liu et al., 2010). Briefly, plants at the four-leaf stage were infiltrated with a 1:1 mixture of TRV1 and TRV2-LIC-*ShORR-1* fragments, and plants treated with cultures with empty vector provided negative controls. Ten days after agro-infiltration, plants were inoculated with *On*. Four plants per trial were inoculated and at least three trials were conducted.

**Table 1:** Names and sequences of primers used in this study.

Primer name	Sequence (5′–3′)	Purpose
*ShORR-1*-F	CGACGACAAGACCCTCCCAATTTTCATAATCCTGTCA	VIGS vector construction of *ShORR-1*
*ShORR-1*-R	GAGGAGAAGAGCCCTCCATTTTGATAAATACCCCTCCA	
*ShORR-1*-F1	ATTACTCTCTTCATAAACTCATTTCCA	Cloning of full-length sequences of *ShORR-1*
*ShORR-1*-R1	TCTGCTGCTATTTCTGCCACT	
*ShORR-1*-F2	ATGTTTGATCCAAGAAAA	Cloning *ShORR-1* from different species
*ShORR-1*-R2	TCAGCAATCTAAATCAGT	
*ShORR-1*-F3	GTCCATGGATGTTTGATCCAAGAAAA	Subcellular localization vector
*ShORR-1*-R3	ATCTAGATCAGCAATCTAAATCAGT	
*ShORR-1*-F4	GTTCATTTCATTTGGAGAGGACAGGGTACCATGTTTGATCCAAGAAAACAAATACCCAA	Construction of *ShORR-1* overexpressing vector
*ShORR-1*-R4	CATTAAAGCAGGGCATGCCTGCAGGTCGACTCAGCAATCTAAATCAGTCATCACTTTGTTTT	
*ShORR-1*-F5	CACCAGCCTTATGGCAAAAT	qRT-PCR analysis
*ShORR-1*-R5	AGTTCCCATTGCCCTCTAGC	
*SlPR1*-F	TGCAACAACGGGTGGTACTT	qRT-PCR analysis
*SlPR1*-R	ATGGACGTTGTCCTCTCCAG	
*SlPR2*-F	CTGCGATGGATCGAACAGGA	qRT-PCR analysis
*SlPR2-*R	TGTGTTGCACCAAAAGCACC	
*SlCOI1*-F	GTAGTCTCGGAGCATCCAGC	qRT-PCR analysis
*SlCOI1*-R	GGGTCCAAAGGCTTGACAGT	
*SlHSR203J*-F	TGGTTCATCAAAAGCAAGTTAAAGA	qRT-PCR analysis
*SlHSR203J*-R	ACCAGTCCATGTCCGGTCTA	
*SlROR2*-F	AGACAAAAGATGGCGTCGGA	qRT-PCR analysis
*SlROR2*-R	TCCTTCACAGCTTCATGCCT	
*SlBI1*-F	GCTCCTCCTTATCAAGAGCAAAA	qRT-PCR analysis
*SlBI1*-R	AGCAGCTGAGAAGCAACCAA	
*SlActin*-F	CCATTCTCCGTCTTGACTTGG	Tomato reference gene
*SlActin*-R	TCTTTCCTAATATCCACGTCAC	

### H_2_O_2_ Accumulation Assay and Microscopic Analysis

An endogenous peroxidase-dependent 3,3-diaminobenzidine (DAB) assay was used to investigate H_2_O_2_ production in plants (Thordal-Christensen et al., 2010). To detect H_2_O_2_ accumulation, leaflets taken 65 h after *On* infection were immersed in DAB solution (1 mg/ml, pH 3.8) for 8–12 h until DAB staining was visible at the vein of the top leaflet. The DAB-stained leaflets were fixed and stained with Coomassie Brilliant Blue R250 in methanol (0.6%, w/v), following Li (2005) with minor modifications. Samples were observed under a differential-interference contrast microscope (Carl Zeiss, Germany), and images were acquired with a Color Video Camera equipped with image analysis software (Image-Pro Plus 4.1, Media Cybernetics, L.P.). In each microscopically examined sample, we observed more than 200 primary haustoria and secondary haustoria, and recorded percentages of host cells showing HR. Three biological replicates of microscopic samples of both *ShORR-1* silenced and control plants were used in these examinations.

### Gene Analog-Based Cloning of *ShORR-1*

RNA was extracted from G1.1560 and MM tomato leaves with TRIzol reagent (Life Technologies, Grand Island, NY) following the manufacturer’s recommendations. After extraction, the RNA samples were treated with DNase I (TaKaRa) to eliminate trace contaminants of genomic DNA. cDNA was synthesized using a PrimeScript RT Perfect Real Time reagent kit (TaKaRa), and the resulting cDNAs were used as templates in the following PCR reactions. The TDF fragment M14E72-213 was aligned to the tomato genome (Consortium, 2012) and ShORR-1-F1/R1 primers (**Table 1**) were designed to clone the complete ORF based on the UniGene with highest identity. To detect genes homologous to *ShORR-1*, sequences from G1.1560 and MM, respectively designated *ShORR-1-G* and *ShORR-1-M*, were amplified with the *ShORR-1*-F2/R2 primers (**Table 1**). The PCR reactions involved denaturation at 94°C for 5 min, followed by 30 amplification cycles of 30 s at 94°C, 45 s at 58°C, 1 min at 72°C, and a final extension step of 10 min at 72°C. The resulting amplicons were inserted into the pMD18-T vector (TaKaRa) and recombinant plasmids were sequenced using the universal T7 primer.

### Transient Expression Vector Construction and Subcellular Localization of *ShORR-1*

ORFs of *ShORR-1-G* and *ShORR-1-M* were used to construct transient expression vectors by insertion into the pSAT6-GFP-N1 vector (Xu et al., 2014), which contains a gene encoding a modified green fluorescent protein (GFP) at *Nco* I–*Xba* I sites, with *ShORR-1*-F3/R3 primers (**Table 1**). pSAT6-GFP-N1 was digested with *Kpn* I and *BamH* I to construct pSAT6-GFP-N1-*ShORR-1-G* and pSAT6-GFP-N1-*ShORR-1-M* vectors, then the recombinant plasmids were transformed into onion epidermal cells by an *Agrobacterium*-mediated *in planta* transient transformation protocol (Xu et al., 2014). Microscopic observations and image acquisition were performed on a fluorescence microscope (Olympus BX61, Japan).

### Generation of Stable Transgenic Plants and Gene Function Verification

To validate functions of *ShORR-1-G* and *ShORR-1-M*, the full length target genes were introduced into the pCAMBIA2300 vector with *Kpn* I and *Sal* I restriction enzymes. Then a 381 bp partial sequence of *ShORR-1-M* was cloned into RNAi vector pCAMBIA2300 using the sense and antisense strands. PCR products were amplified with KAPA HiFi PCR kits (Kapa Biosystems, USA), then transformed into *Escherichia coli* Trans T1 competent cells to generate recombinant plasmids, which were introduced into *Agrobacterium* strain GV3101. All applied oligonucleotide primers are listed in **Table 1**. Different homozygotes transgenic plants lines were produced by the Plant Genetic Transformation Center of the Henan Key Laboratory of Crop Molecular Breeding & Bioreactor. To investigate *ShORR-1*’s function in resistance to *On*, the positive transgenic plants were inoculated and microscopically analyzed, as previously described (Shen et al., 2007). Fresh leaves from wild-type and transgenic tomato plants were collected and placed on 1% agar plates containing 85 µM benzimidazole. The leaves were incubated in a climate chamber providing constant light at 20°C for at least 4 h, then inoculated with a suspension of *On* spores (5 × 10^3^ conidia/ml). After allowing the *On* fungus to develop on the leaves for 65 h under the same conditions (Bai et al., 2005), the leaves were fixed in ethanol/acetic acid (1:1, v/v), stained with Coomassie Brilliant Blue R250 in methanol (0.6%, w/v) for 10 s, then rinsed in deionized water. Samples were subsequently observed with a BX61 microscope (Olympus, Tokyo, Japan), and microcolonies were counted. More than 1,000 germinated spores on each leaf segment from every plant were observed, and three biological replicates of control and transgenic plants were used.

### Quantitative Reverse Transcription PCR

To quantify levels of *ShORR-1* transcript produced in response to *On*, susceptible and resistant tomato leaves were sampled at 0, 8, 24, 36, 72, and 120 h postinoculation (hpi), according to previous microscopic observations of tomato-*On* interaction (Gao et al., 2014). Estimates of fungal biomass in the samples were obtained by extracting *On* DNA and quantifying levels of the *EF-1a* gene following Trond and Cathrine (2009). To explore the resistance mechanisms involving *ShORR-1*, the expression of six marker genes associated with different disease resistance and hormone pathways was quantified in wild-type and *ShORR-1-G*-overexpressing plants in the presence and absence of *On*. For this, total RNA was extracted from samples of transgenic and wild-type plants with TRIzol reagent following recommendations of the manufacturer, and 1 µg total RNA was used for cDNA synthesis. It was then subjected to quantitative reverse transcription PCR (qRT-PCR) using a CFX96™ Real-Time PCR Detection System (Bio-Rad, Hercules, CA) with SYBR^®^ Premix Ex Taq™ (Takara Bio Inc., Shiga, Japan). The amplification conditions consisted of 95°C for 3 min, followed by 40 cycles of 95°C for 10 s, and 60°C for 30 s. Fold changes in levels of target transcripts were calculated using the 2^−ΔΔCt^ and 2^−ΔCt^ method (Livak and Schmittgen, 2001), with normalization against the tomato *Actin* (*SlActin*) transcript levels. Three biological and technical replicates were run for each cDNA sample. All the applied qRT-PCR primers are listed in **Table 1**.

### Data Analysis

Relative expression levels of genes were statistically analyzed using one-way analysis of variance (ANOVA) and Dunnett’s *post hoc* or Tukey’s HSD tests (*P* < 0.01 and *P* < 0.05). All analyses were performed using SPSS Statistics 17.0 following instructions in the SPSS Survival Manual.

## Results

### Cloning and Sequence Analysis of *ShORR*-*1* in Resistant and Susceptible Tomato Genotypes

**Figure 1:**
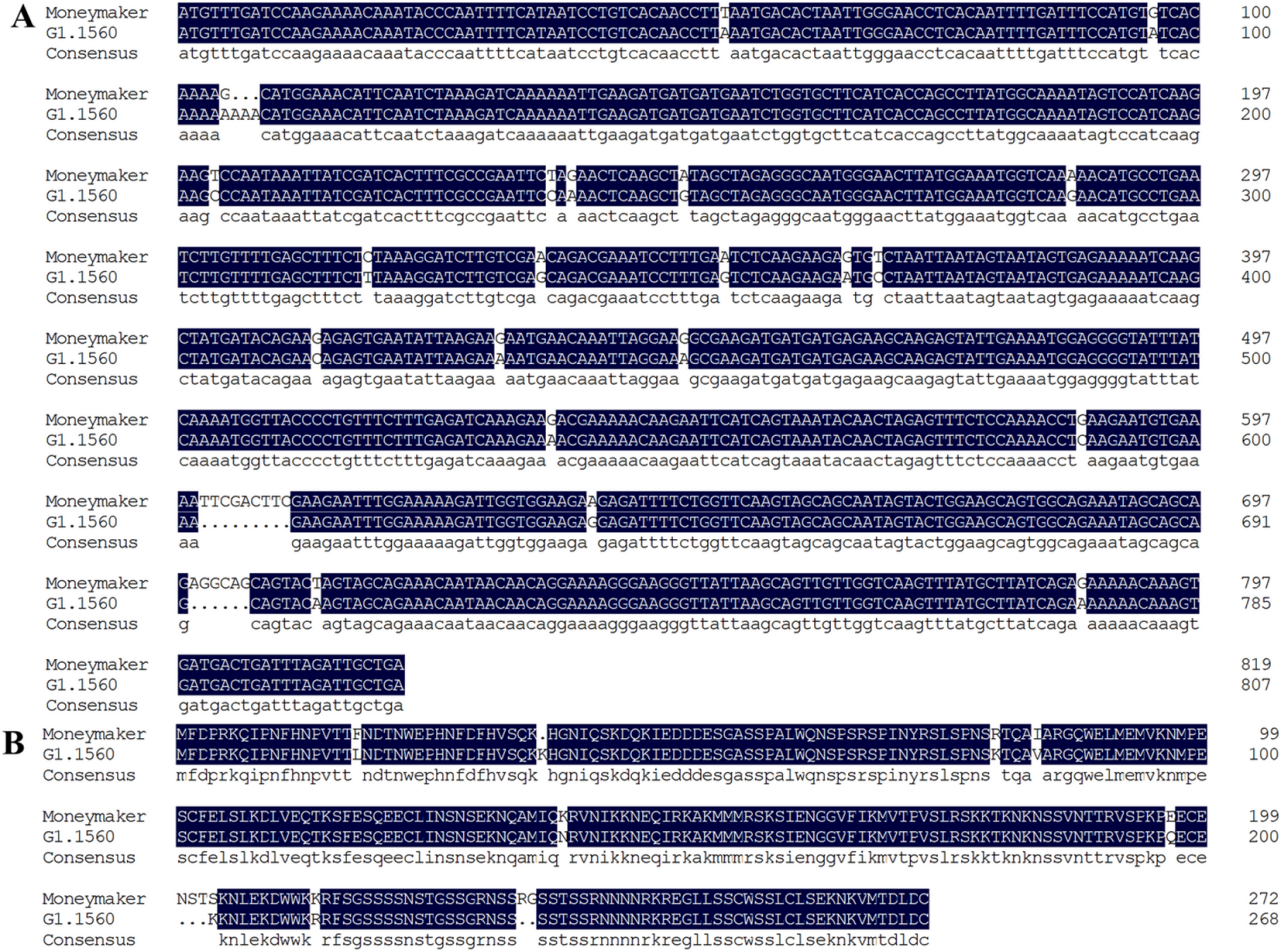
Nucleotide **(A)** and amino acid **(B)** sequences alignment of ShORR-1-M and ShORR-1-G share high homology using DNAMAN software.

In our previous cDNA-AFLP study, we found that TDF fragment M14E72-213—designated No. 25 by Li et al. (2007), Appendix 1)—was present in *On*-resistant NIL-*Ol-1*, but not in the *On*-susceptible cultivar MM. BLAST analysis indicated that the 134 bp sequence had been annotated in SGN before the tomato genome sequence became publicly available, and was described as having 97% identity with a UniGene of unknown function (SGN-U319851). Using the sequence of this UniGene, in the study presented here we cloned the 807 bp ORF designated *ShORR-1-G* from the *Ol-1* resistant line G1.1560 (accession no. MK205292). We found it encodes a putative protein of 268 amino acids with a molecular weight of 30.55 kDa, isoelectric point (pI) of 9.53, and 95% identity to a protein of unknown function encoded by Solyc06g059860.2 according to a BLASTP search against the SGN database. In contrast, the ORF sequence of *ShORR-1-M* is 819 bp long (accession no. MK205293), and encodes a putative protein of 272 amino acids with a molecular weight of 30.96 kDa, isoelectric point (pI) of 9.48, and 99% identity with Solyc06g059860.2 (differing in only one nucleotide base). *ShORR-1-G* and *ShORR-1-M* have no conserved domain, according to a search of the NCBI database. The DNAMAN software package was applied to align the nucleotide and protein homologs of ShORR-1 cloned from the two tomato varieties (**Figure 1**). The ORF and protein sequence homologies of the susceptible and resistant species’ ShORR-1 variants were 95.26 and 94.87%, respectively. The alignment results showed that ShORR-1-G has one more amino acid residue (a lysine) than ShORR-1-M at the 5′ end, while there are five more amino acids in ShORR-1-M at the 3′ end, and seven other amino acids differ between them. Additionally, the amino acid sequences of ShORR-1-G and ShORR-1-M shared 95.22 and 97.42% identity with a hybrid signal transduction histidine kinase of *S. pennellii* with a BLASTP search in NCBI, respectively. The ShORR-1-G and ShORR-1-M sequences respectively contain 19 and 20 potential serine phosphorylation sites (http://www.dabi.temple.edu/disphos), and in both cases putative MAPK phosphorylation sites (Mao et al., 2011, Nobuaki et al., 2011) in the N terminus (**Figure S1**). Moreover, ShORR-1-M and ShORR-1-G proteins both have potential ubiquitination (**Figure S2**) and SUMOylation sites (**Figure S3**) by UbPred (http://www.ubpred.org/) and GPS-SUMO (http://sumosp.biocuckoo.org/online.php) (Radivojac et al., 2010; Zhao et al., 2014). Neither ShORR-1-M and ShORR-1-G proteins have any transmembrane helices according to TMHMM 2.0 predictions (http://www.cbs.dtu.dk/services/TMHMM/).

### ShORR-1 Localized in Plasma Membrane

**Figure 2:**
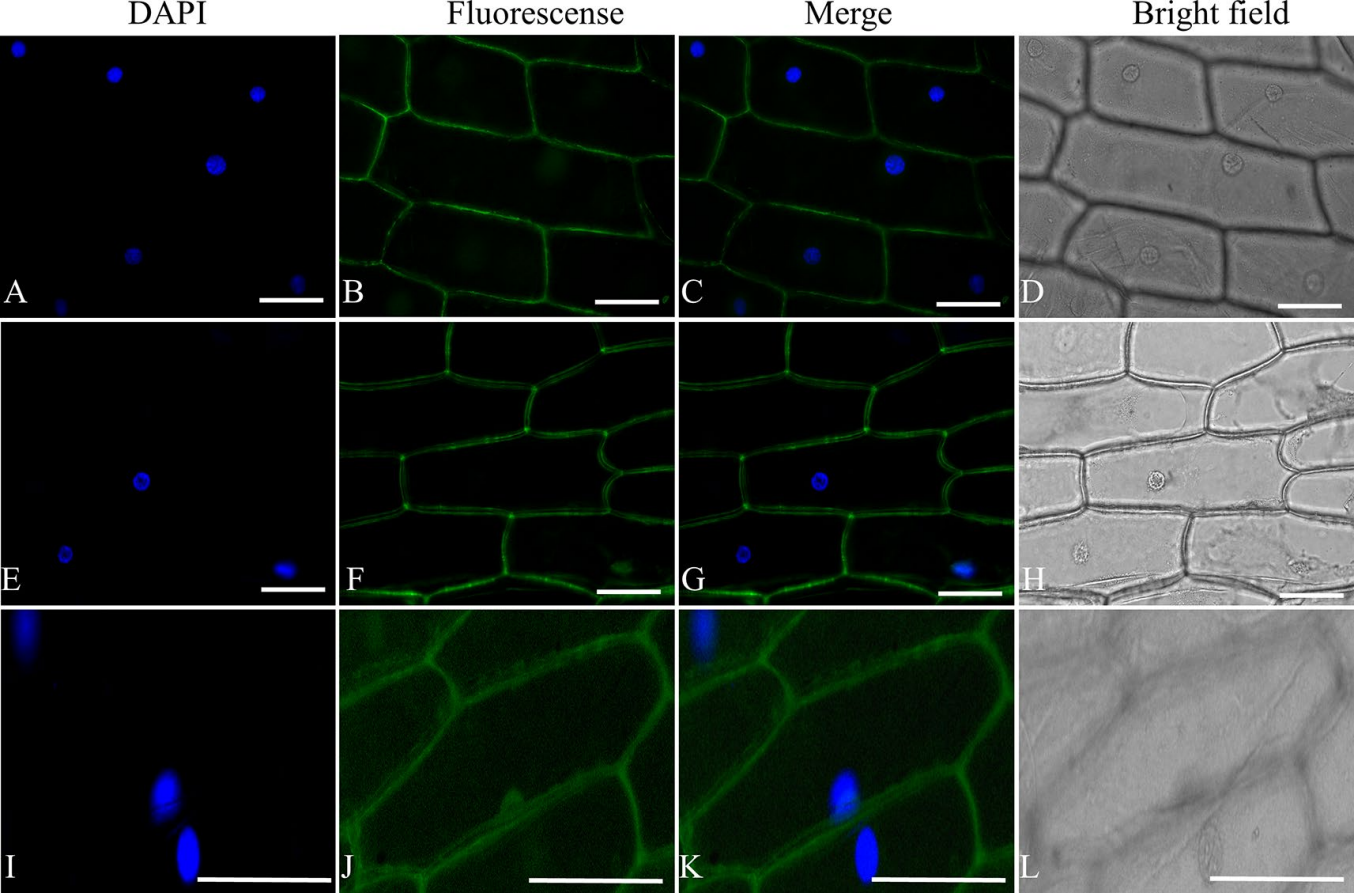
ShORR-1-M and ShORR-1-G both localized to the plasma membrane. Subcellular localization GFP fusions with ShORR-1 variants isolated from G1.1560 and MM in onion epidermal cells, as shown by 4′,6-diamidino-2-phenylindole (DAPI) nuclear staining **(A**, **E**, and **I)**, green fluorescent protein (GFP) fluorescence **(B**, **F**, and **J)**, merger of DAPI staining and fluorescence **(C**, **G**, and **K)**, bright field microscopy **(D**, **H**, and **L)**. The proteins displayed in panels **(A**–**D)**, **(E**–**H)**, and **(I**–**L)** were transiently expressed using pSAT6-GFP-N1-ShORR-1-G, pSAT6-GFP-N1-ShORR-1-M, and a construct designed to express GFP alone (as a control), respectively. Scale bar = 25 µm.

To determine the subcellular localization of ShORR-1, the full-length sequences isolated from G1.1560 and MM were fused to the 5′ terminus of the *GFP* gene in the pSAT6-GFP vector, under control of the constitutive CaMV: 35S promoter. The ShORR-1 protein was transiently transferred into onion epidermal cells by *A. tumefaciens*-mediated transformation (Xu et al., 2014). Microscopic observation of the fluorescent signal emitted by the ShORR-1-GFP fusion protein revealed that ShORR-1-M and ShORR-1-G were both localized on the plasma membrane, while fluorescence signals from the control pSAT6-GFP vector were ubiquitous in examined cells (**Figure 2**). These results show that ShORR-1 is a membrane-localized protein.

### Expression Pattern of *ShORR-1* in Tomato

**Figure 3:**
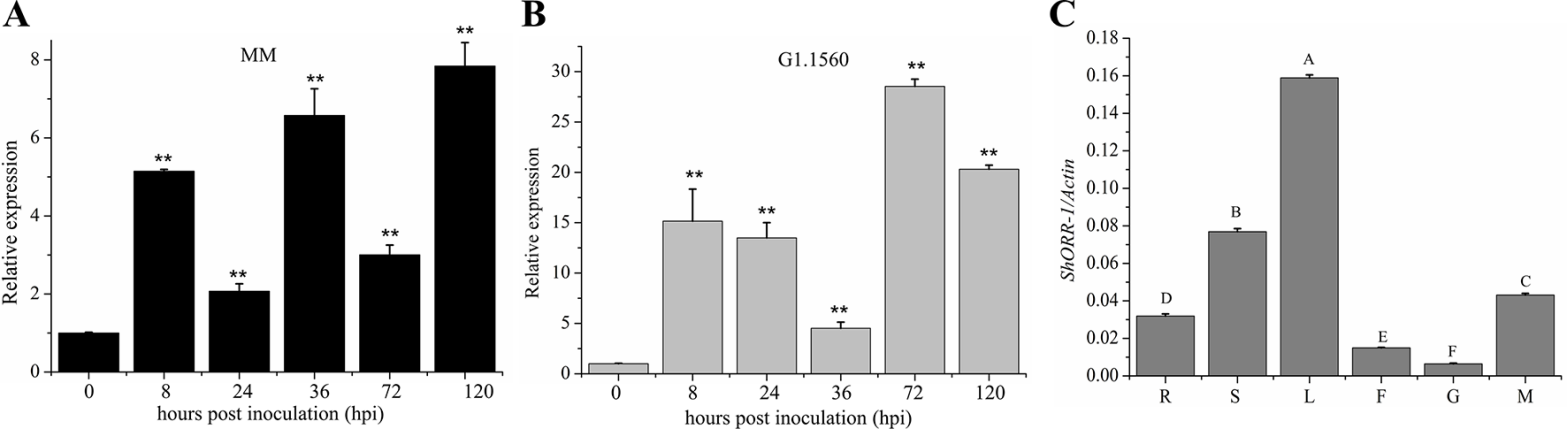
Expression profiles of *ShORR-1* at indicated *On* infection stages and tissues in MM **(A)** and G1.1560 **(B)**. Samples were harvested at 0, 8, 24, 36, 72, and 120 hpi **(A**–**B)**. The relative expression levels of *ShORR-1* at each time-point were normalized relative to *SlActin*. Asterisks indicate significant difference from the control determined by one-way ANOVA followed by an independent-samples Dunnett’s *post hoc* test (**: *P* < 0.01). **(C)** The transcript accumulation of *ShORR-1* was examined by quantitative real-time PCR from MM tissues, including roots (R), stems (S), leaves (L), flowers (F), green fruits (G), and red fruits (M). *Actin* was used as an internal control. Mean and standard error were calculated using data from three independent biological replicates. Letters indicate significant differences between different tissues determined by one-way ANOVA followed by Tukey’s HSD test (*P* < 0.01).

To investigate *ShORR-1*’s role in resistance to infection by the powdery mildew fungus *On*, we measured its transcript levels in tissues of leaves infected by *On* at selected time-points by qRT-PCR. Previous research had revealed that *On* haustoria form at 24–41 hpi (Huang et al., 1998; Bai et al., 2005). Expression of *ShORR-1* was detected in both *On*-susceptible and -resistant species, but the transcription patterns and expression levels (relative to levels in controls) differed. Generally, relative expression levels of *ShORR-1* were much higher in G1.1560 than in MM, although they were similar at 36 hpi (**Figures 3A, B**). The relative expression level of *ShORR-1* in MM increased as early as 8 hpi, and peaked (at 7.8-fold) at 120 hpi, compared to levels in control leaves (**Figure 3A**), while the relative expression level of *ShORR-1* in G1.1560 also increased at 8 dpi and peaked at 72 hpi (**Figure 3B**). These results indicate that *ShORR-1* could be upregulated earlier and generally more strongly in resistant tomato plants than in susceptible tomato plants in response to *On*. Quantitative analysis of *ShORR-1* expression patterns in MM tissues by qRT-PCR showed that it is expressed mainly in leaves and stems, and to a lesser extent in mature red fruits, roots, flowers, and green fruits (**Figure 3C**).

### Loss of Resistance to Powdery Mildew in *ShORR-1*-Silenced Tomato Leaves

**Figure 4:**
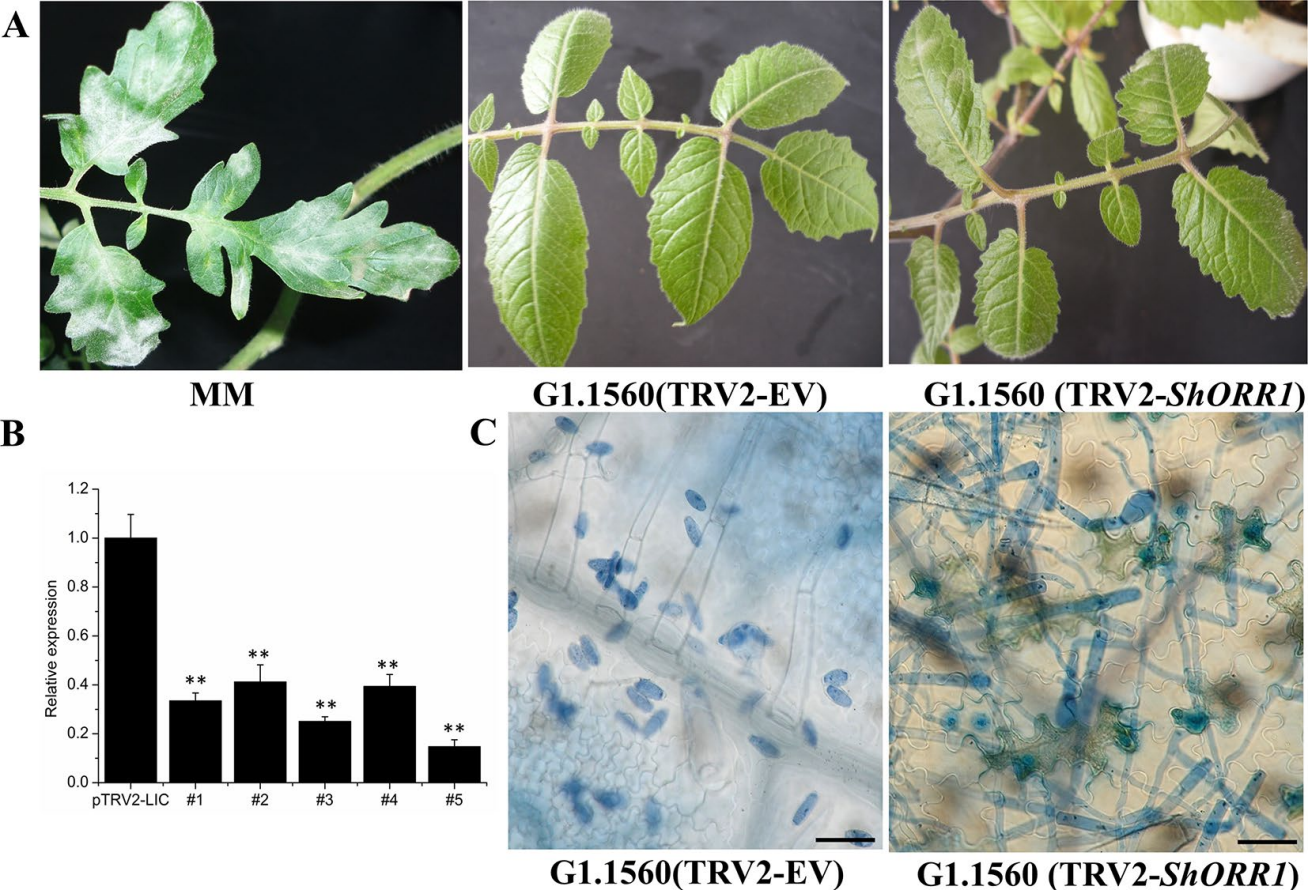
Downregulation of *ShORR-1* by VIGS compromises *Ol-1*-mediated resistance. **(A)** *On* susceptible phenotypes of MM and *ShORR-1-*silenced (TRV2-*ShORR-1*) G1.1560 plants, and resistant phenotype of G1.1560 controls transformed with an empty vector (TRV2-EV). **(B)** Levels of *ShORR-1* transcripts in control and TRV2-*ShORR-1* leaves. Error bars represent standard deviations, obtained from analysis of three biological replicates. Asterisks indicate significant difference from the control by one-way ANOVA followed by an independent-samples Dunnett’s *post hoc* test (**: *P* < 0.01). **(C)** Microscopic morphologies of *On* in control and TRV2-*ShORR-1* plants. Bar = 25 µm.

For the TDF fragment M14E72-213, which had 97% identity with SGN-U319851, primers were designed based on U319851 to construct a TRV2-VIGS vector to silence *ShORR-1* in *S. habrochaites* G1.1560. Twenty silenced plants of this previously resistant line were inoculated with *On* 10 days after VIGS infiltration. Seven days postinoculation (dpi) with the fungus, *On* colonies were clearly visible on leaves of all 20 silenced plants, with 5–30% of their foliar area affected, while no fungal colonies were observed on plants infiltrated with the empty vector (TRV2-EV) (**Figure 4A**). These results clearly indicate that silencing of *ShORR-1* abolished *Ol-1*-mediated resistance to *On* at the macroscopic level. qRT-PCR analyses showed that *ShORR-1* transcript levels were significantly lower in the silenced plants than in controls (**Figure 4B**). Moreover, microscopic analysis showed that numerous conidiophores with conidia were present on *ShORR-1*-silenced resistant plants, and the histological morphology of the fungus on *ShORR-1*-silenced resistant plants was similar to that on susceptible plants. Thus, the fungus successfully completed its life cycle on them, and silencing of *ShORR*-*1* did not lead to morphological alteration of *On*. In contrast, fungal growth was clearly prevented, and no conidiospores formed, on TRV2-EV G1.1560 plants (**Figure 4C**). In conclusion, silencing *ShORR-1* allowed fungal growth and sporulation, resulting in visible disease symptoms, indicating that *ShORR-1* plays an important role in resistance to powdery mildew caused by *On*.

### H_2_O_2_ Accumulation Analysis, *On* Growth and Host Response

**Figure 5:**
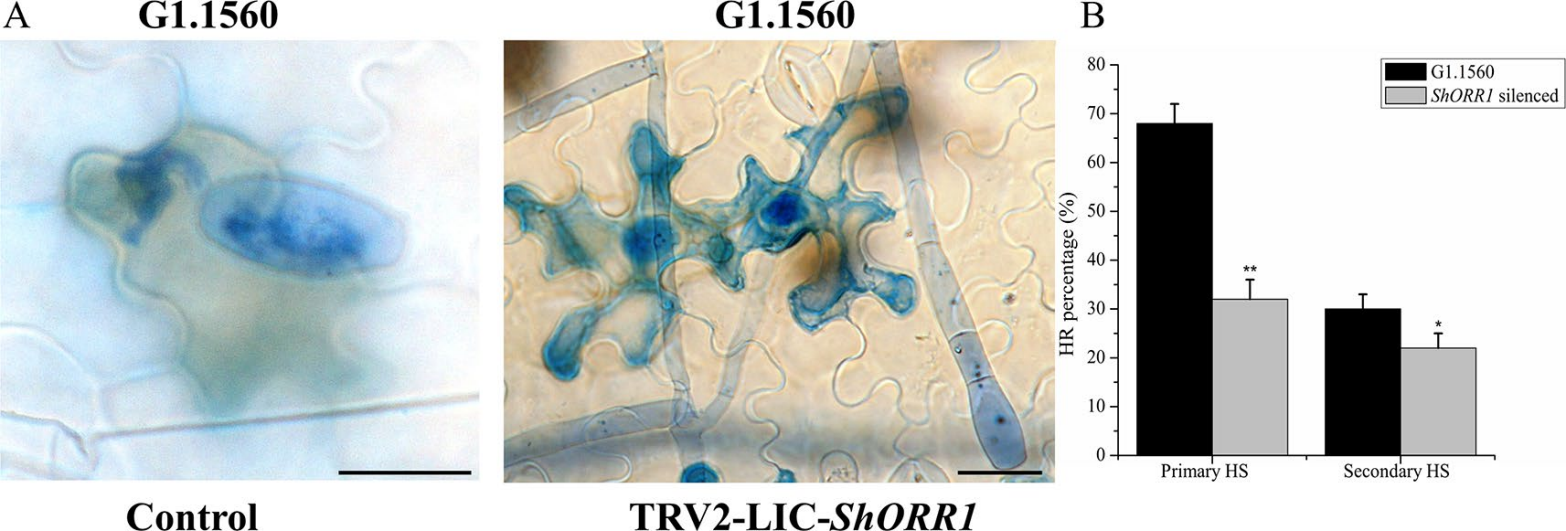
*ShORR-1*-silenced plants show decreased *On*-induced HR formation and H_2_O_2_ accumulation. **(A)** Micrographs of resistant *S. habrochaites* G1.1560 without *ShORR-1* silencing (left) and *ShORR-1*-silenced *S. habrochaites* G1.1560 (right). Bar = 15 µm. **(B)** Percentages of HR-associated primary and secondary haustoria (HS) in *ShORR-1*-silenced and G1.1560 control plants. Asterisks indicate significant difference from the control by one-way ANOVA followed by an independent-samples Dunnett’s *post hoc* test (**: *P* < 0.01, *: *P* < 0.05). Three biological replicates of microscopic samples of both *ShORR-1* silenced and control plants were observed.

Li (2005) noted that the TDF fragment M14E72-213 accumulated more rapidly, and to higher levels, in resistant tomato than in susceptible plants after inoculation with *On*. Microscopic analysis presented here showed that *S. habrochaites* G1.1560 control plants displayed a rapid HR following exposure to *On*. In this response, most cells invaded by primary haustoria of *On* rapidly accumulated H_2_O_2_ and died, thereby preventing further growth of *On* (**Figure 5A**). Contrary to expectations, *On*-induced cell death and H_2_O_2_ accumulation were observed in *ShORR-1*-silenced resistant plants (**Figure 5A**), although fungal growth and conidiophore formation were similar to those on susceptible plants. However, the proportion of dead cells among cells attacked by fungal haustoria was lower in *ShORR-1*-silenced resistant plants than in control resistant plants. We investigated more than 200 cells attacked by primary haustoria in each microscopic sample, and found that average percentages of cells showing HR in control and *ShORR-1*-silenced plants were about 68 and 30%, respectively, at 64 hpi. We then observed more than 200 cells attacked by secondary haustoria in each microscopic sample, and found that percentages of cells showing HR in control and *ShORR-1*-silenced plants were about 32 and 22%, respectively, at 147 hpi (**Figure 5B**). In contrast to those in resistant control plants, most cells invaded by fungal haustoria remained alive in *ShORR-1*-silenced resistant plants, and the haustoria showed normal morphology, resulting in further fungal growth and conidiophore formation in *ShORR-1*-silenced resistant plants. The results also indicated that the *On*-induced HR in the *ShORR-1*-silenced resistant plants was slower, and possibly weaker, than in the control resistant plants.

### Overexpressing of *ShORR-1-G*, But Not *ShORR-1-M*, Enhanced Resistance to *On*

**Figure 6:**
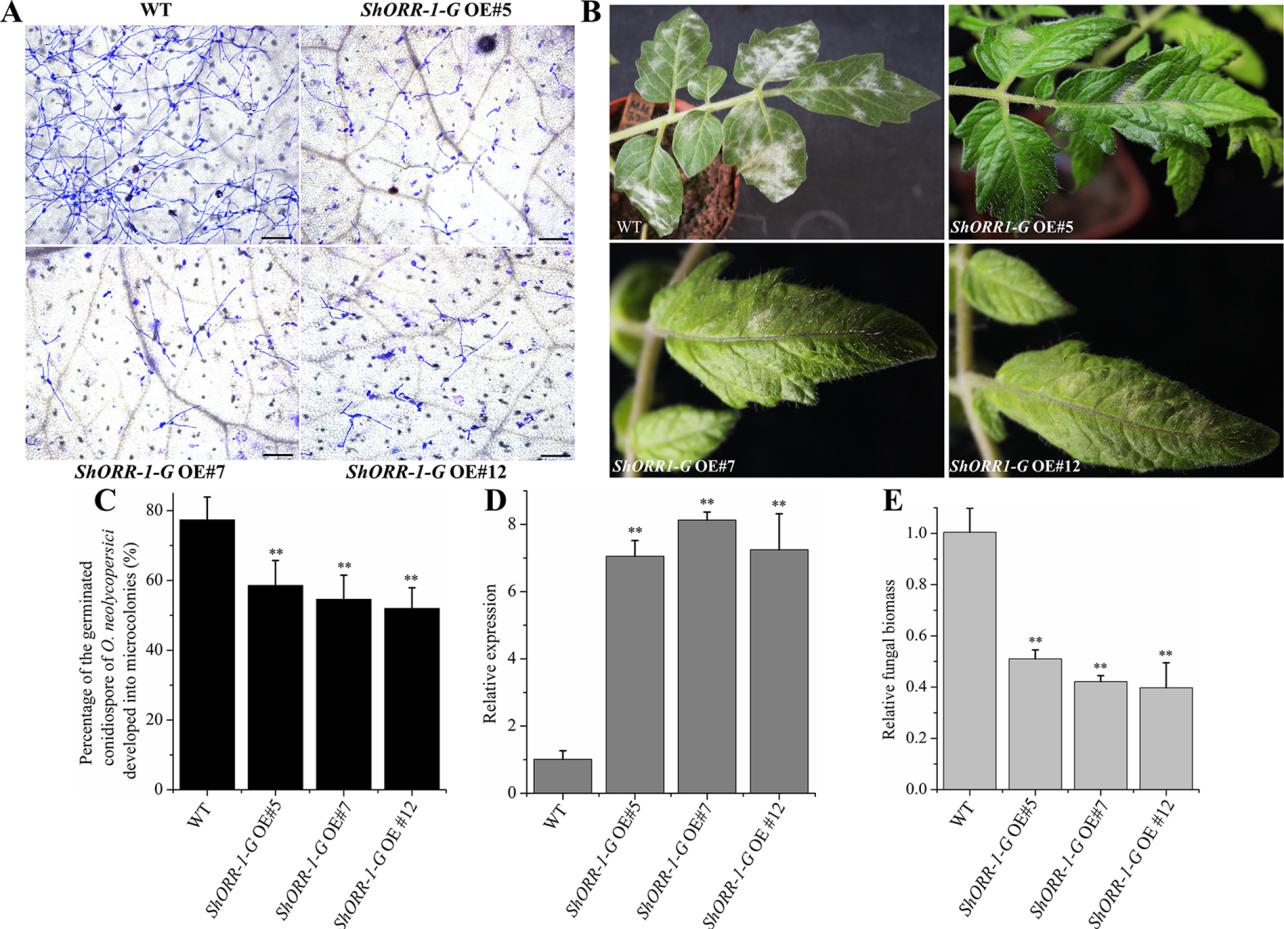
Overexpressing of *ShORR-1-G* enhanced tomato plants’ resistance to *On*. **(A)** Micrographs of powdery mildew on leaves of T2 *ShORR-1-G* overexpressing plants and untransformed MM plants, 65 h after inoculation. Scale bar = 25 µm. **(B)** Macroscopic phenotypes of *On* infected leaves of untransformed MM plants and T2 *ShORR-1-G* overexpressing plants. **(C)** Percentages of germinated *On* conidiospores on untransformed MM plants and T2 *ShORR-1-G* overexpressing plants at 65 h after infection. **(D)** Levels of *ShORR-1* transcripts in three transformed plants and controls. Double asterisks indicate significant differences from the control by one-way ANOVA followed by an independent-samples Dunnett’s *post hoc* test (*P* < 0.01). **(E)** Estimated *On* fungal biomass on control plants and three transformed lines. All the above results are based on analyses of three biological replicates of control and transgenic plants.

To further investigate the role of *ShORR-1-G* in resistance to *On*, stable RNAi and overexpressing MM transformants of *ShORR-1-G* and *ShORR-1-M* were generated. Samples collected at 65 hpi were microscopically examined, and percentages of germinated *On* conidiospores that developed into microcolonies on them were recorded. There were significantly fewer *On* microcolonies on leaves of transgenic plants overexpressing *ShORR-1-G* than on leaves of wild-type plants (**Figures 6A, C**). In addition, numerous clear mycelial colonies were macroscopically observed on leaves of control plants, while few were found on leaves of *ShORR-1-G* overexpressing plants (**Figure 6B**). The efficiency of gene overexpressing was confirmed by qRT-PCR analyses, which showed that *ShORR-1-G* transcript levels were 6.5-fold higher in the overexpressing lines than in control plants (**Figure 6D**). Moreover, fungal biomass was 4- to 5-fold lower on the *ShORR-1-G* overexpressing plants, according to genetically based estimates (**Figure 6E**).

**Figure 7:**
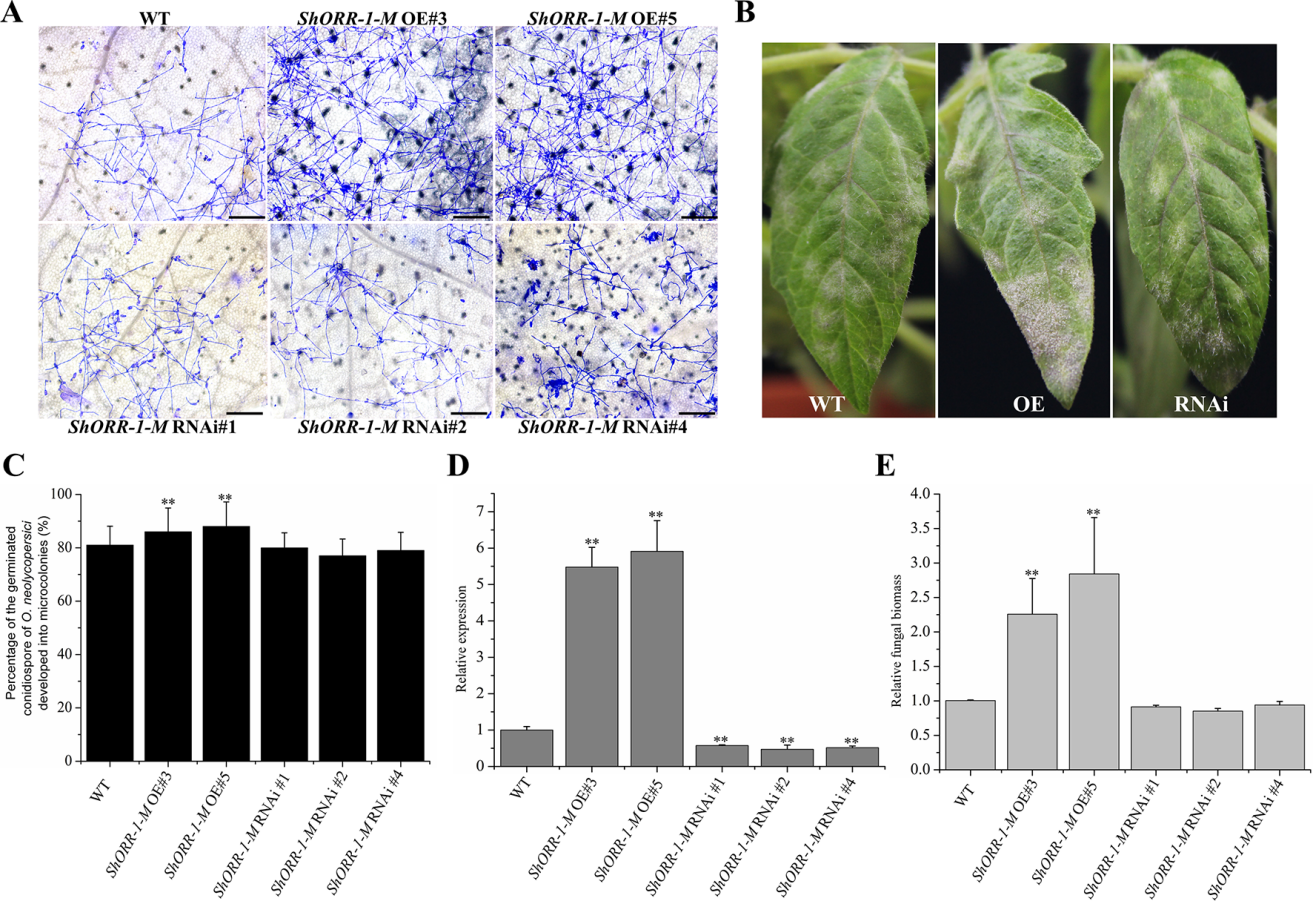
Overexpressing of *ShORR-1*-*M* increased susceptibility to *On*, but silencing it did not increase resistance to *On*. **(A)** Micrographs of powdery mildew on leaves of T2 *ShORR-1*-*M* overexpressing and silenced plants, compared with untransformed MM plants, 65 h after inoculation. Scale bar = 25 µm. **(B)** Macroscopic phenotypes of *On* infected leaves of T2 *ShORR-1*-*M* overexpressing and silenced plants. **(C)** Percentages of germinated *On* conidiospores on *ShORR-1*-*M* overexpressing and silenced plants, 65 h after infection. **(D)** Levels of *ShORR-1* transcripts in transgenic plants and controls. Double asterisks indicate significant differences from the control by one-way ANOVA followed by an independent-samples Dunnett’s *post hoc* test (*P* < 0.01). **(E)** Estimated *On* fungal biomass on transgenic and control plants. All the above results are based on analyses of three biological replicates of control and transgenic plants.

However, microscopic observation indicated that overexpressing *ShORR-1-M* transgenic plants were more susceptible to *On* than control plants, while the plants with silenced *ShORR-1-M* had similar susceptibility to the controls (**Figures 7A, C**). Macroscopic observation confirmed these findings (**Figure 7B**). The gene silencing and overexpressing levels were confirmed by qRT-PCR analyses, which showed that *ShORR-1-M* transcript levels were dramatically lower in the gene-silenced plants and higher in the overexpressing transgenic plants than in the controls (**Figure 7D**). Quantification of fungal biomass confirmed that *ShORR-1-M* overexpressing increased the plants’ susceptibility, but silencing of *ShORR-1-M* did not increase resistance to *On* (**Figure 7E**). These results clearly indicate that *ShORR-1-G* is required for *Ol-1* mediated resistance to *On* in tomato plants, while its homolog *ShORR-1-M* promotes susceptibility to *On*, presumably due to the differences in their sequences.

**Figure 8:**
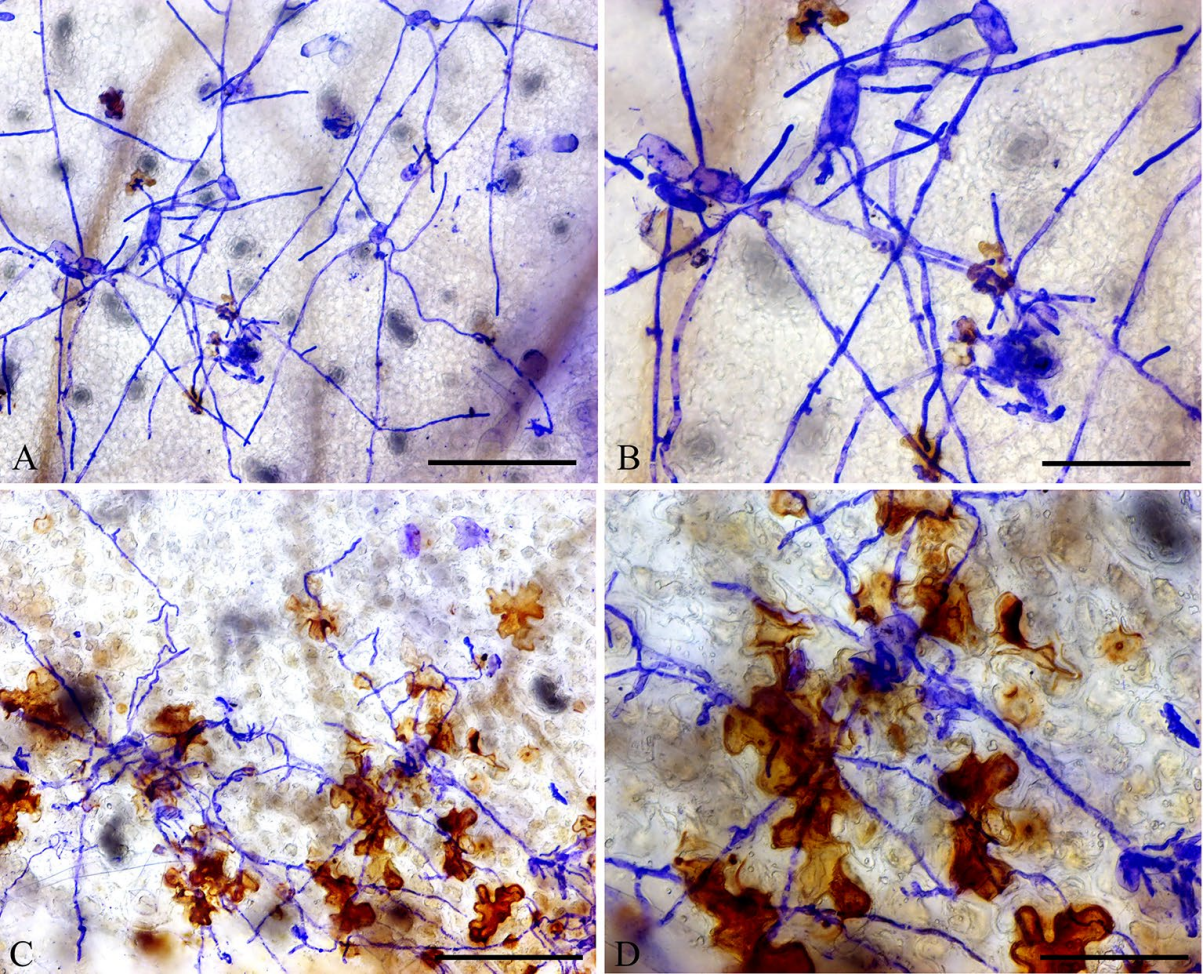
Overexpressing of *ShORR-1-G* increased H_2_O_2_ accumulation in transgenic tomatoes. **(A)** Wild-type tomato leaves were inoculated with *On* and sampled 65 hours after infection. **(B)** Enlarged view of A. **(C)** *ShORR-1-G* overexpressing tomato leaves challenged with *On* and sampled 65 h after infection. **(D)** Enlarged view of **(C)**, showing H_2_O_2_ accumulation in epidermal cells, as manifested by 3,3-diaminobenzidine (DAB) staining. Scale bars = 25 µm in **(A** and **C)**, 12.5 µm in **(B** and **D)**.

**Figure 9:**
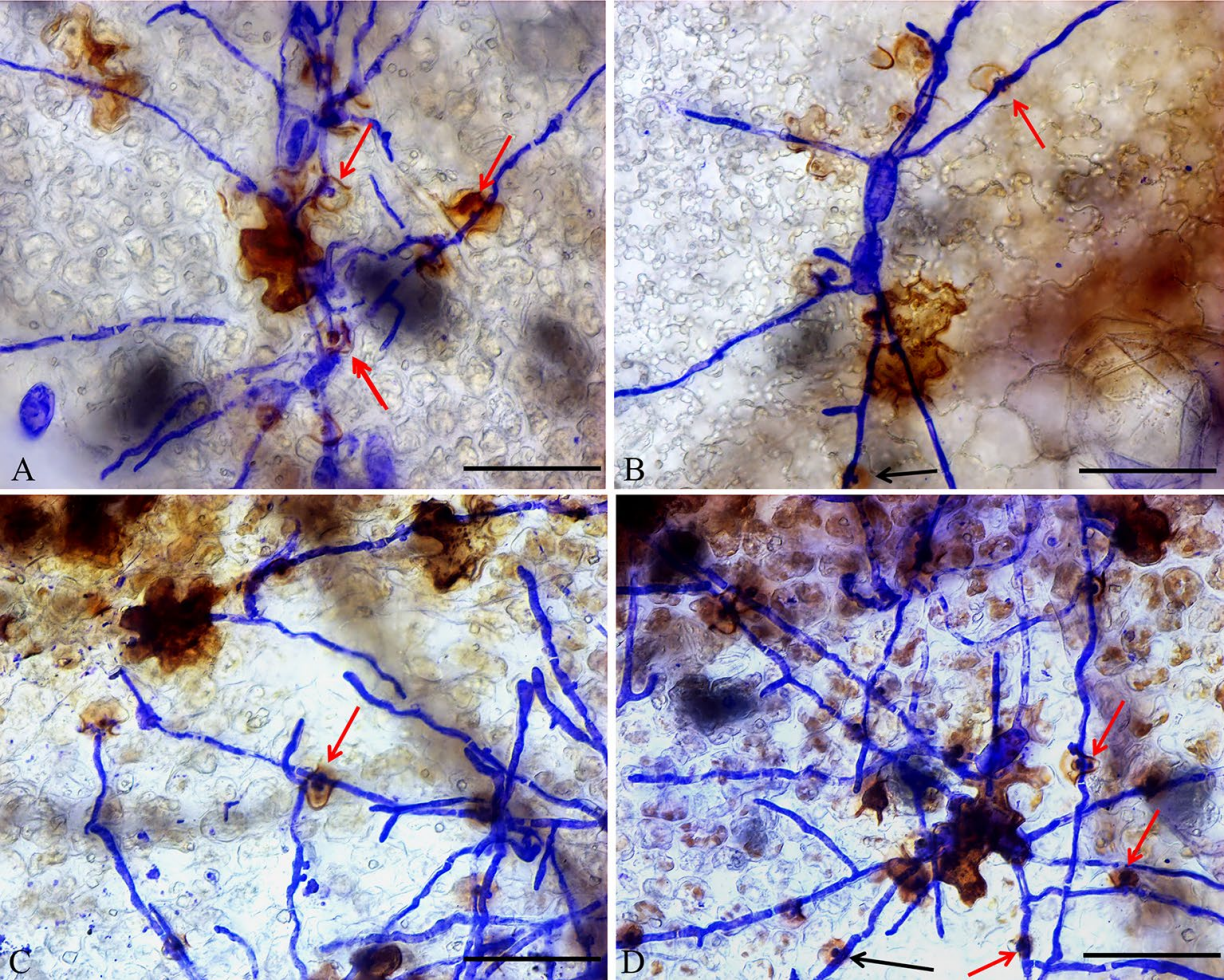
Micrographs of haustorium phenotypes in *ShORR-1-G* overexpressing plants. Red and black arrows show morphologically abnormal and normal haustoria with high H_2_O_2_ contents, respectively. Scale bar = 12.5 µm. Panels **A**–**D** show the different haustorium phenotypes in *ShORR-1-G* overexpressing lines 5, 7, 12 and 13.

In addition, DAB staining revealed that H_2_O_2_ levels were higher in epidermal cells of *ShORR-1-G* overexpressing plants than in those of wild-type plants at 65 hpi with *On* (**Figure 8**). Thus, *ShORR-1-G* positively regulates resistance to *On* in tomato, and the resistance is associated with H_2_O_2_ accumulation. Moreover, upregulation of *ShORR-1-G* resulted in more abnormal haustoria (**Figure 9**, red arrows) in attacked cells than in untransformed plants. These haustoria were irregular and oval, had high H_2_O_2_ contents, and some seemed to be plasmolyzed (**Figure 9B**).

### Overexpressing of *ShORR-1* Triggered or Suppressed Expression of Six Resistance Marker Genes

**Figure 10:**
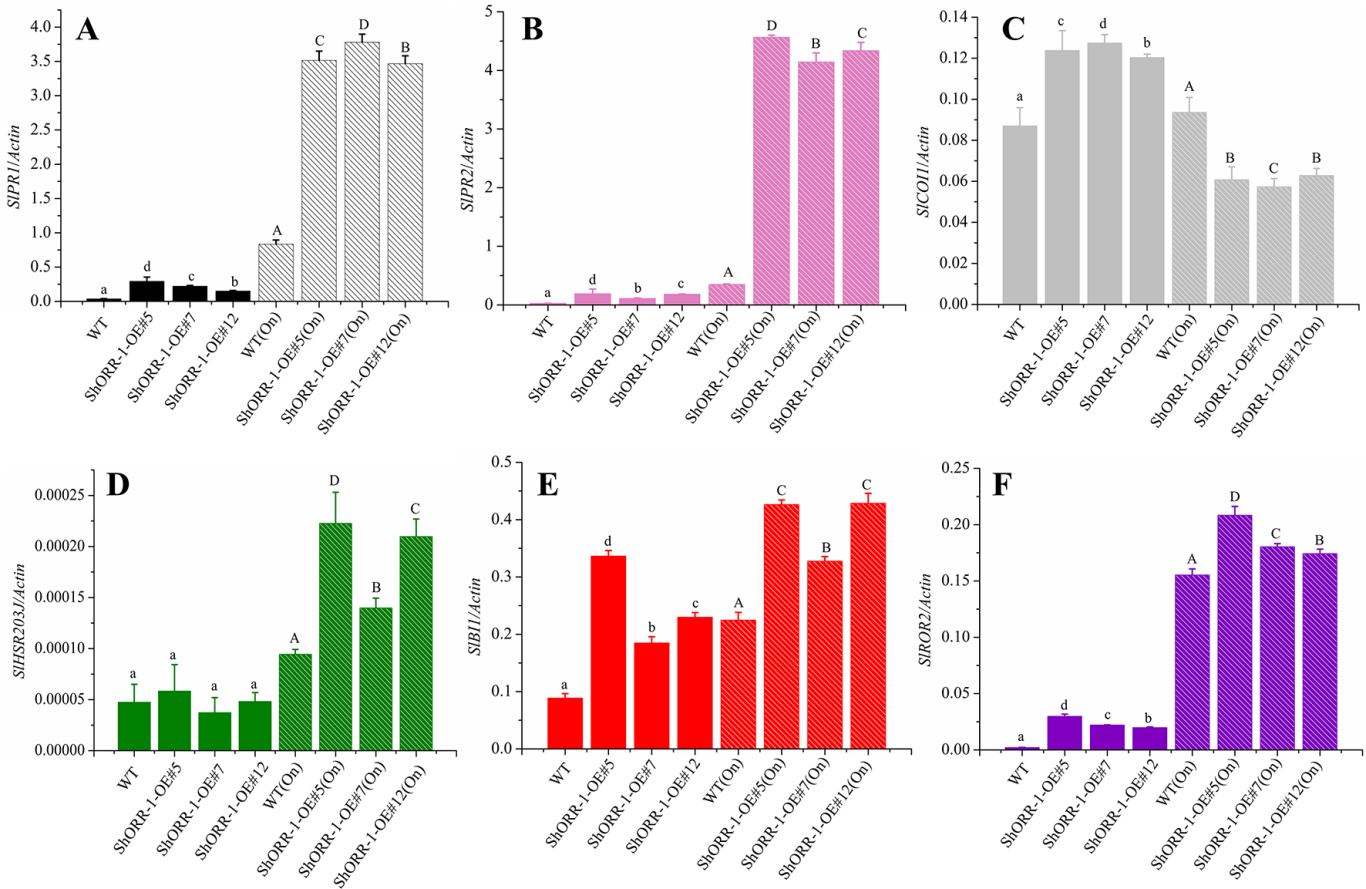
Quantitative real-time PCR analysis of expression levels of the following selected defense marker genes in wild-type and *ShORR-1-G* overexpressing (OE) transgenic tomatoes with and without *On* infection: **(A)** *SlPR1*, **(B)** *SlPR2*, **(C)** *SlCOI1*, **(D)** *SlHSR203J*, **(E)** *SlROR2*, **(F)** *SlBI1*. *Actin* was used as an internal control. Value for each sample is mean of three biological replicates. Mean and standard error were calculated using data from three independent biological replicates. Lowercase letters were used to indicate significant differences between lines in untreated (without *On* infection) conditions, and capital ones indicate significant differences between lines after *On* infection. Upper and lowercase letters indicate significant differences relative to control (WT) plants determined by one-way ANOVA followed by Tukey’s HSD test (*P* < 0.05).

Induced plant defenses are regulated by a highly interconnected signaling network in which the plant hormones jasmonic acid (JA) and salicylic acid (SA) play central roles (Pieterse et al., 2009, Pieterse et al., 2012). To investigate pathways that may be recruited in defenses against powdery mildew in *ShORR-1*-*G* overexpressing tomato plants, we used qRT-PCR to analyze the expression of six defense genes involved in JA (*COI1*) and SA (*PR1*, *PR2*) pathways, HR (*HSR203J*, *BI1*) and other important aspects of disease resistance (*ROR2*). Three *ShORR-1*-*G* overexpressing transgenic lines and MM were chosen to probe expression levels of these six marker genes in the presence and absence of *On* infection. The qRT-PCR results revealed that SA, JA, and HR might be involved in tomato resistance to *On* mediated by *ShORR-1* (**Figure 10**).

*PR1* and *PR2* (encoding β-1,3-glucanase; also called *BGL2*) have been widely used as molecular marker genes for the SA hormone pathway in plants (Glazebrook, 2005; López-Cruz et al., 2017; Yang et al., 2018; Miao et al., 2019; Wang et al., 2019). Our results showed that expression levels of *SlPR1* and *SlPR2* were higher in *On*-infected wild-type plants and *ShORR-1-G* overexpressing plants than in wild-type plants without *On* infection. Furthermore, *SlPR1* and *SlPR2* expression levels were dramatically higher (350- and 400-fold, respectively) in *On*-infected transgenic plants. Thus, powdery mildew infection dramatically induced expression of *SlPR1* and *SlPR2*, and overexpressing of *ShORR-1* enhanced this induction, in accordance with previous findings that the powdery mildew *Erysiphe orontii* can elicit accumulation of *PR1* and *PR2* in *Arabidopsis* (Lynnereuber et al., 2010). However, *On* inoculation did not significantly affect transcript levels of *SlCOI1*, encoding coronatine insensitive 1, a key component of JA-mediated defense pathways (Li et al., 2004), in wild-type plants, while it substantially suppressed *SlCOI1* expression in *ShORR-1-G* overexpressing plants. Moreover, we did not detect significant difference in expression levels of *SlHSR203J* and *SlBI1*—two important HR marker genes that are highly and rapidly induced in plant defense responses to various bacterial and fungal pathogens (Sanchez et al., 2000, Pontier et al., 2001)—between wild-type and *ShORR-1-G* overexpressing transgenic plants. However, *SlBI1* and (especially) *SlHSR203J* were induced more strongly by *On* in *ShORR-1-G* transgenic plants than in wild-type plants, and their expression levels correlated with the extent of HR. Required for *mlo*-specified resistance (*ROR2*), an essential component of *mlo*-mediated basal penetration resistance to barley powdery mildew, has a specialized resistance function that is conserved between monocotyledons and dicotyledons (Collins et al., 2003). We found that *SlROR2* was significantly induced in the *ShORR-1-G* overexpressing plants, with and without *On* inoculation, suggesting *ShORR-1-G* overexpression triggered this basal resistance.

## Discussion

Several DE-TDFs between powdery mildew-resistant NILs and the susceptible cultivar MM have been previously identified, including M14E72-213 (Li et al., 2006, Li et al., 2007). In the study presented here we found that M14E72-213 has high homology to a previously uncharacterized gene, which we named *ShORR-1*. By VIGS and overexpressing approaches, we demonstrated that a resistant variant of *ShORR-1* is essential for *Ol-1*-based resistance (**Figures 4** and **6**). Moreover, its overexpressing is associated with H_2_O_2_ accumulation (**Figure 8**) and abnormal haustoria (**Figure 9**), and hence enhances the resistance.

### *ShORR-1* Is a Novel and Indispensable Gene in *Ol-1*-Mediated Tomato Resistance to *On*

Silencing of *ShORR-1* in the resistant tomato cultivar *S. habrochaites* G1.1560 resulted in a susceptible phenotype, as shown by whole plant disease assays and microscopic analysis of *On*-infected plants (**Figure 4**). Downregulation of *ALS* and *ShGST*, two other DE-TDFs induced in NIL-*Ol-1*, both compromised tomato’s resistance to powdery mildew caused by *On*, indicating that multiple resistance genes are involved in *Ol-1*-mediated resistance to *On* (Pei et al., 2011a; Gao et al., 2014).

We also found that silencing *ShORR-1-G* attenuated defense responses, i.e. rapid HR and H_2_O_2_ accumulation, which are normally induced strongly by infection with *On* in G1.1560 plants (**Figure 5**). The weakened responses (lacked by susceptible plants) were not sufficient to prevent growth and development of *On*. Similarly, knock-down of *ShGST* in G1.1560 tomato plants reduced resistance to *On*, and resulted in slow rather than rapid HR (Pei et al., 2011a). This response differed from the slow HR of the NIL-*Ol*-*1*, developed by backcrossing *S. habrochaites* G1.1560 with MM, in which cells invaded by primary fungal haustoria remained alive, and only cells invaded by secondary haustoria died (Li et al., 2006).

Furthermore, overexpressing of *ShORR-1*-*G* increased the *On* resistance of susceptible MM plants (**Figure 6**), and resulted in higher levels of H_2_O_2_ in their epidermal cells upon *On* attack than in wild-type plants (**Figure 8**). HR, which is frequently observed around the infection site when microbial pathogens attack, arrests development of the pathogen at the epidermal cell attacked (Perfect and Green, 2001; Salguero-Linares and Coll, 2019). H_2_O_2_ production closely accompanies HR, and is believed to be an important diffusive signal in programmed cell death. Our results suggest that *ShORR-1*-*G* (or another resistant variant of *ShORR-1*) is vital for resistance to powdery mildew caused by *On* in tomato, since its overexpressing enhanced HR, H_2_O_2_ levels and resistance to *On* infection, whereas there was less H_2_O_2_ accumulation in the highly susceptible MM. Moreover, in *ShORR-1*-*G* overexpressing plants we examined, and NIL-*Ol-1* plants examined by Li (2005), more abnormal haustoria were observed than in wild-type counterparts (**Figure 9**). Haustoria are generally fungal pathogens’ main structures for nutrient uptake (Roman-Reyna and Rathjen, 2017) and signal exchange (especially delivery of virulence effectors) with host plants (Hacquard et al., 2013). Our results suggest that the haustorial abnormalities in *ShORR-1-G* overexpressing plants at least partially prevented the fungus from absorbing nutrients and water from the host, thus reducing the extent of its growth and infection. However, in the NIL-*Ol-1* plants the abnormal haustoria were reportedly filled with small vesicles, which were not observed in the plants we examined, although abnormal plasmolyzed haustoria were found in both NIL-*Ol-1* (Li, 2005) and *ShORR-1* overexpressing plants. These findings suggest that the *Ol-1* and *ShORR-1* resistance mechanisms may have both similarities and differences.

### *ShORR-1* Variants in MM and G1.1560 Have Antagonistic Effects in Responses to *On*

M14E72-213 DE-TDF was found in NIL*-Ol-1*, but not either NIL-*Ol-4* or MM plants (Li et al., 2006, Li et al., 2007). However, ORFs of *ShORR-1* were isolated from both *On*-resistant NIL-*Ol-1* and *On*-susceptible MM plants (**Figure 1**), with mutations at 13 amino acid sequence sites. It indicated the above contrary was caused by the following, sequence analysis of ShORR-1-M and ShORR-1-G suggested that the mutation between two homologs at the 234th nucleotide base of ShORR-1-M was just at the annealing sequence region of selective primer E72 designed based on the recognition site of restriction enzyme (*EcoR* I) and selective nucleotide, sequence analysis also indicated that E72 can anneal at digested fragments of ShORR-1-G but not at those of ShORR-1-M (Li, 2005), which resulted in the DE-TDF of M14E72-213 was only identified in *ShORR-1-G* but not in *ShORR-1-M* in the cDNA-AFLP analysis using M14 (designed based on the recognition site of restriction enzyme (*Mse* I) and selective nucleotide) and E72 selective primer combination. Functional analysis showed that overexpressing of *ShORR-1-M* increased susceptibility to *On*, while *ShORR-1-M*-silenced plants phenotypically resembled untransformed controls (**Figure 7**). All the results indicate that *ShORR-1*-*G* plays a vital role in resistance to *On*, but not *ShORR*-*1*-*M*. Similarly, the *R* gene *PigmR* confers broad-spectrum resistance (*inter alia* to the blast fungus *Magnaporthe oryzae* in rice), while *PigmS* (which differs in four amino acids) competitively attenuates *PigmR* homodimerization to suppress resistance (Deng et al., 2017). Whether the mutation sites in resistant lines play important roles in *ShORR-1*-mediated *On* resistance needs to be verified by site-specific mutation in future studies. In addition, sequences of ShORR-1-G and ShORR-1-M proteins respectively contain 20 and 19 serine phosphorylation sites (**Figure S1**), including in both cases a cluster of four potential MAPK phosphorylation sites (Ser-57, Ser-64, Ser-68, Ser-76) in the N terminus (**Figure S1**). Thus, ShORR-1 could be phosphorylated by MPKs, but this also requires verification.

### JA, SA, HR, and Basal Resistance Are Involved in *ShORR-1*-Mediated Resistance to Powdery Mildew

Plants are not passive victims when attacked by microbial pathogens. SA-dependent signaling plays significant roles in plant resistance to biotrophic pathogens, especially powdery mildew, while the JA signaling pathway is important in resistance to necrotrophic pathogens, but there is complex crosstalk between the JA and SA signaling pathways (Glazebrook, 2005). When a plant is attacked by a biotrophic pathogen, this crosstalk leads to activation of the SA defense pathway and inhibition of the JA signaling pathway (Fu and Dong, 2013). We observed the same patterns in tomato plants infected by the powdery mildew *On*, especially *ShORR-1-G* overexpressing plants, in which the SA-related defense genes *SlPR1* and *SlPR2* were strikingly activated, but the JA-related defense gene *SlCOI1* was repressed. Various biotrophic pathogens have evolved intricate mechanisms that enable them to evade plant defenses by hijacking the JA pathway (Yan and Xie, 2016). Moreover, increasing evidence indicates that they inject effectors and toxins into plant cells that prevent the triggering of host defenses by targeting JA pathway components (Cole et al., 2014, Gimenez-Ibanez et al., 2014). Whether *ShORR-1* is associated with the SA- and JA-dependent pathways requires validation in further studies, for example by overexpressing *ShORR-1-G* in tomato lines with perturbances in the pathways, and/or screening for proteins that interact with ShORR-1 to elucidate the pathway(s) that ShORR-1 influences.

When attacked by fungal pathogens, HR is one of the most effective resistance responses (Salguero-Linares and Coll, 2019). The HR marker gene *SlHSR203J* is reportedly activated in tomato plants by the leaf mold pathogen *Cladosporium fulvum* (Pontier et al., 1998), and (in both susceptible and resistant tomato taxa) following exposure to *On* (Li, 2005). Our results confirmed that *On* significantly induced expression of *SlHSR203J*, accompanied by increases in numbers of epidermal cells displaying hypersensitive responses (**Figure 9**).

Another important gene in powdery mildew resistance is *Mildew Locus O* (*MLO*). Recessive loss-of-function (*mlo*) mutations in the gene confer resistance to powdery mildew in barley (Miklis et al., 2007), tomato (Bai et al., 2008), pepper (Zheng et al., 2013), pea (Pavan et al., 2011), wheat (Várallyay et al., 2012), and *Arabidopsis* (Consonni et al., 2006). The resistance mechanism is based on early abortion of fungal pathogenesis, with formation of papillae at attempted penetration sites (Bai et al., 2005). *ROR2* plays a major role in *mlo*-mediated disease resistance, and the barley *ror2* mutant reportedly has less penetration resistance but stronger HR than wild-type plants when attacked by barley powdery mildew (Collins et al., 2003). We found that both *On* infection and *ShORR-1*-*G* overexpressing induced expression of *SlROR2*, accompanied by higher frequencies of cells showing HR, especially in *ShORR-1*-*G* overexpressing tomatoes challenged by *On*. These findings suggest that upregulation of *SlROR2* might lead to formation of papillae, resulting in enhancement of resistance to *On*, but this hypothesis requires further validation.

In summary, *ShORR-1* (originally detected in the form of a DE-TDF in our previous cDNA-AFLP analysis) plays an essential role in *Ol-1*-mediated resistance. *ShORR-1-G* and *ShORR*-*1*-*M*, respectively cloned from G1.1560 and MM, have antagonistic roles in responses to *On*, presumably due to differences (13) in their amino acid sequences. Future research will decipher roles of mutation sites and analysis of the relationship of *ShORR-1* and *Ol-1* might provide new insights into the mechanisms of *Ol-1-*mediated tomato resistance to powdery mildew.

## Data Availability Statement

The datasets generated for this study can be found in the Genbank.

## Author Contributions

CL conceived and designed the research. YZ, KX, DP, GC, HY, and WZ performed the experiments. DY, JZ, and XL provided fungal materials, reagents, and analytical tools. YZ and DP analyzed the data, prepared figures, and wrote the paper. All authors read and reviewed the final manuscript.

## Funding

This research was supported by the National Natural Science Foundation of China (grant nos. 31902030, 31571997, 31872129, 31071807, and 31272168), Natural Science Foundation of Henan province (grant no. 182300410058), Foundation of Henan Science and Technology Committee (grant nos. 192102110001 and 192102110124), and Training Program of Youth Backbone Teacher of Henan Province (grant no. 2017GGJS146).

## Conflict of Interest

The authors declare that the research was conducted in the absence of any commercial or financial relationships that could be construed as a potential conflict of interest.

## Supplementary Material

Figure S1 Potential serine phosphorylation sites in ShORR-1-G **(A)** and ShORR-1-M **(B)** were predicted by DISPHOS (Version 1.3). The blue circles indicate putative MAPK phosphorylation sites in ShORR-1.

Figure S2 Potential lysine ubiquitination sites of ShORR-1-G **(A)** and ShORR-1-M **(B)** were predicted by UbPred and graphed by IBS (Version 1.0). The threshold of confidence was set to be more than 0.69, and U indicates ubiquitination sites.

Figure S3 Potential lysine SUMOylation sites of ShORR-1-G **(A)** and ShORR-1-M **(B)** were predicted by GPS-SUMO 2.0 Online Service and graphed by IBS (Version 1.0). Medium stringency (*Ac*> 89.12%, *Sn*> 67.93%, *Sp*> 90%, *MCC*> 0.3446, *Pr*> 21.96%) was chosen as the threshold for GPS-SUMO prediction, and S indicates SUMOylation sites.
