# Supplementary figures and images for: ShORR-1, a Novel Tomato Gene, Confers Enhanced Host Resistance to Oidium neolycopersici
Source: Front Plant Sci. 2019 Nov 7;10:1400. doi: 10.3389/fpls.2019.01400 (PMC6854008; doi:10.3389/fpls.2019.01400)

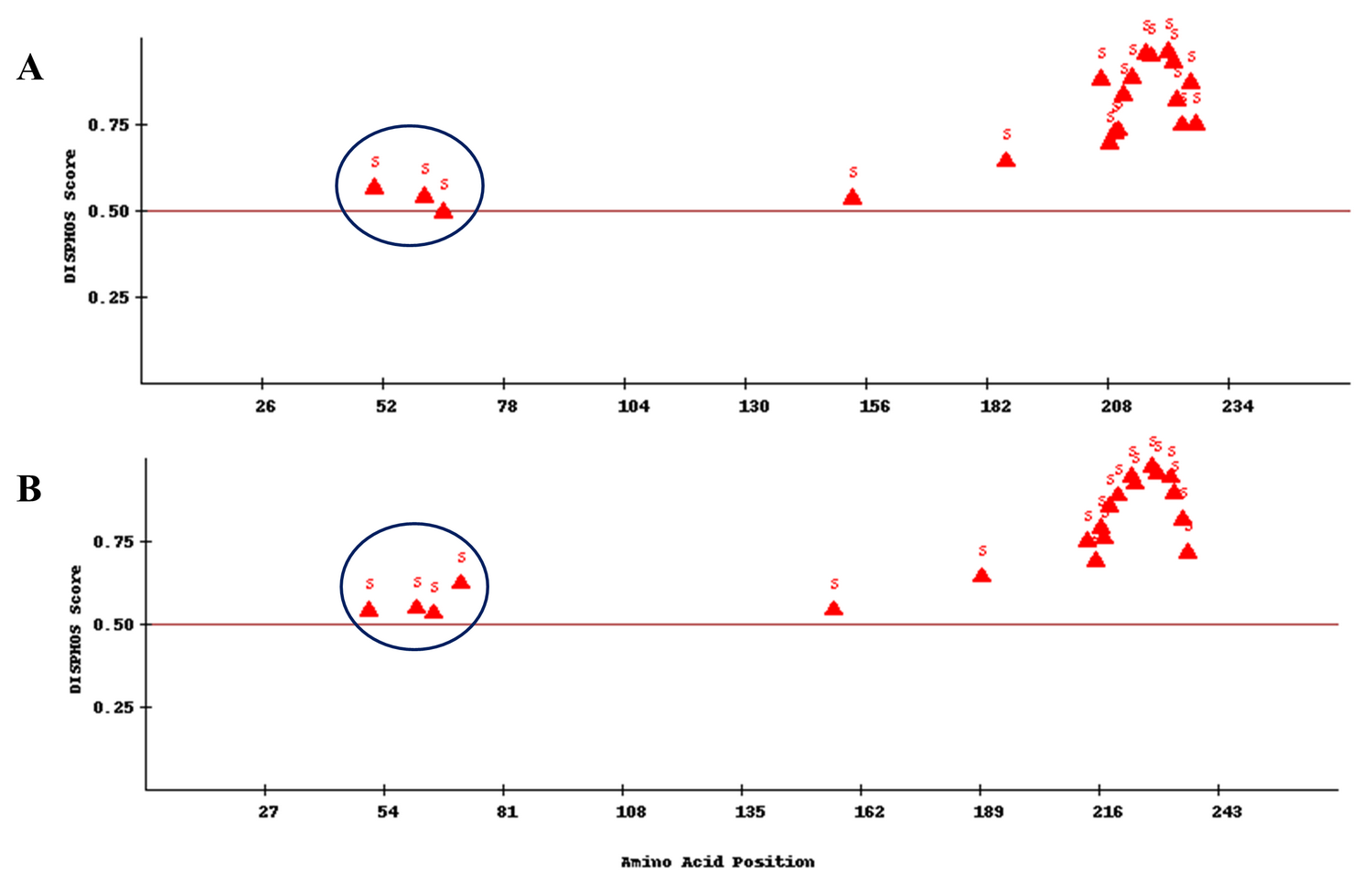

Supplement: Figure S1 — Potential serine phosphorylation sites in ShORR-1-G (A) and ShORR-1-M (B) were predicted by DISPHOS (Version 1.3). The blue circles indicate putative MAPK phosphorylation sites in ShORR-1. [file Image_1.tif]

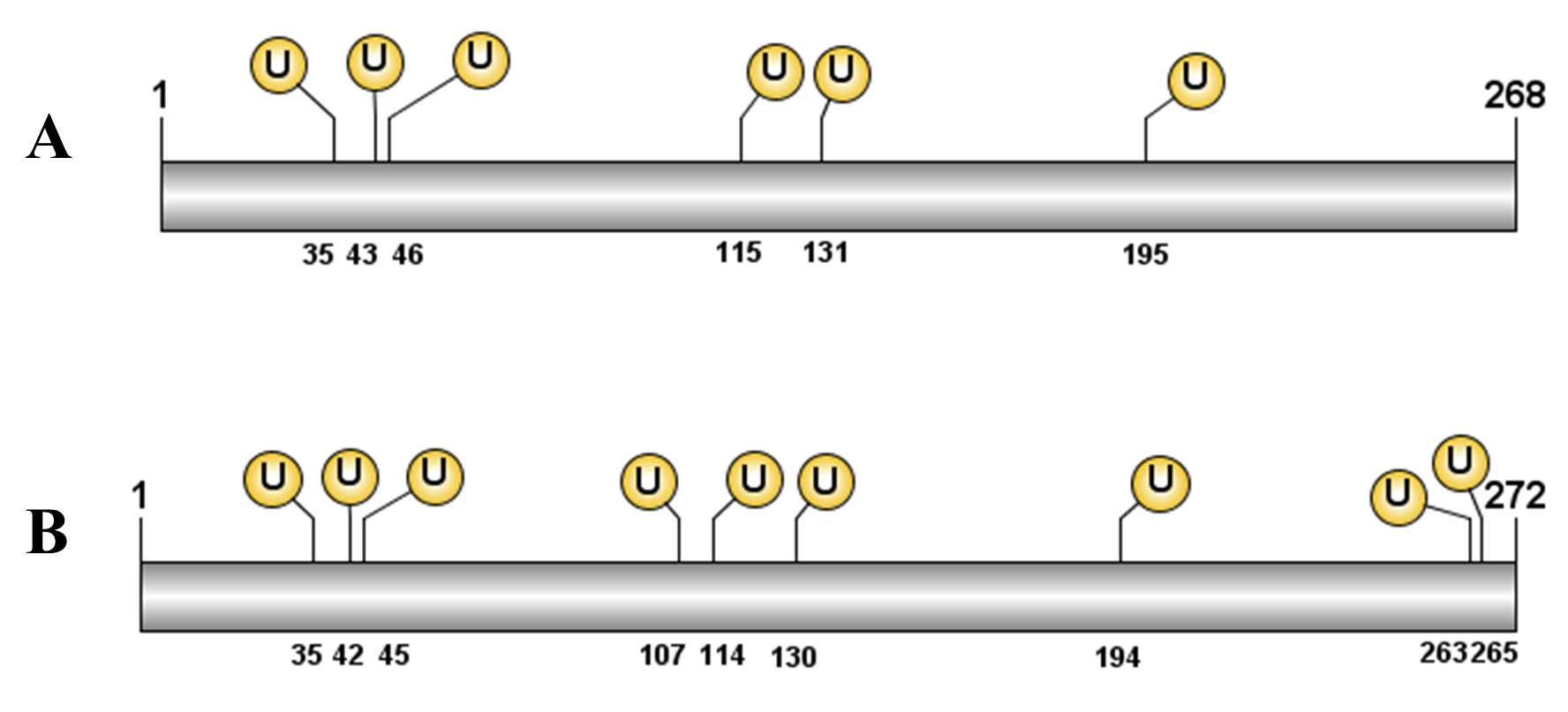

Supplement: Figure S2 — Potential lysine ubiquitination sites of ShORR-1-G (A) and ShORR-1-M (B) were predicted by UbPred and graphed by IBS (Version 1.0). The threshold of confidence was set to be more than 0.69, and U indicates ubiquitination sites. [file Image_2.tif]

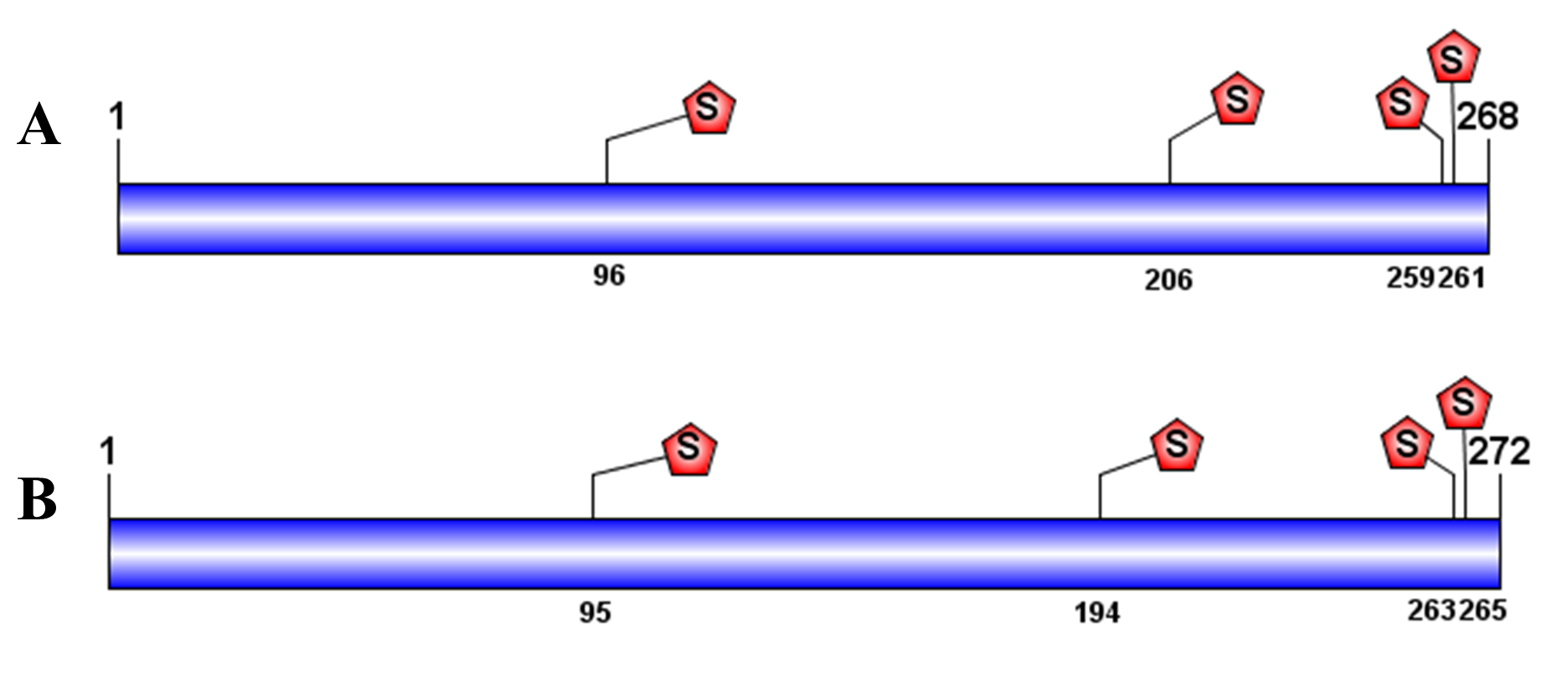

Supplement: Figure S3 — Potential lysine SUMOylation sites of ShORR-1-G (A) and ShORR-1-M (B) were predicted by GPS-SUMO 2.0 Online Service and graphed by IBS (Version 1.0). Medium stringency (Ac> 89.12%, Sn> 67.93%, Sp> 90%, MCC> 0.3446, Pr> 21.96%) was chosen as the threshold for GPS-SUMO prediction, and S indicates SUMOylation sites. [file Image_3.tif]
